# Vegan and omnivore diets in relation to nutrient intake and greenhouse gas emissions in Iceland

**DOI:** 10.1038/s41598-025-03193-3

**Published:** 2025-05-25

**Authors:** Ragnhildur Gudmannsdottir, Steina Gunnarsdottir, Emese Kenderesi, Holmfridur Thorgeirsdottir, Johanna Eyrun Torfadottir, Ingibjorg Gunnarsdottir, Inga Thorsdottir, Amanda Wood, Olof Gudny Geirsdottir, Bryndis Eva Birgisdottir, Thorhallur Ingi Halldorsson

**Affiliations:** 1 Faculty of Food Science and Nutrition, School of Health Science, University of Iceland, Saemundargata 12, Reykjavik, 102 Iceland; 2 Unit for Nutrition Research, University of Iceland, Landspitali University Hospital, Reykjavik, Iceland; 3 Directorate of Health, Reykjavik, Iceland; 4 Centre of Public Health Sciences, School of Health Science, University of Iceland, Reykjavik, Iceland; 5 Stockholm Resilience Center, Stockholm University, Stockholm, Sweden; 6 Centre for Fetal Programming, Department of Epidemiology Research, Statens Serum Institut, Copenhagen, Denmark; 7 Department of Nutrition, Exercise and Sports, University of Copenhagen, Copenhagen, Denmark

**Keywords:** Vegans, Omnivores, Nutrition, Sustainability, Greenhouse gas emissions, Carbon footprint, Computational biology and bioinformatics, Environmental sciences, Biomarkers

## Abstract

Plant-rich diets have gained recognition for their environmental sustainability. However, relatively few studies have compared dietary habits of vegans and omnivores in terms of compliance with nutritional recommendations and dietary greenhouse gas (GHG) emissions, which this study aimed to assess. We used data on 651 omnivores and 68 vegans from the Icelandic National Dietary Survey (2019–2021) and a comparable survey on vegans (2022–2023), respectively. The median dietary GHG emissions was substantially lower among vegans than omnivores (2.6 vs. 5.3 kg CO_2_-eq/day). Compared to omnivores, vegans had a proportionally higher intake of energy from carbohydrates (48% vs. 39%) but a lower intake from fat (35% vs. 40%) and proteins (12% vs. 18%). More vegans compared to omnivores fell within the dietary recommendations for fiber (74% vs. 8%) and saturated fat (56% vs. 7%) while vegans were less likely to have protein intake in line with the recommended ≥ 0.83 g/kg body weight (52% vs. 79%). Despite frequent use of dietary food supplements among vegans (97%) and omnivores (72%) the prevalence of those reaching the recommended intake of iodine, calcium and vitamin D, was low (40–60%) in both groups. In conclusion, vegans were overall more compliant with macronutrient recommendations and had substantially lower dietary GHG emissions compared to omnivores. In terms of meeting dietary recommendations, room for improvements was observed in both groups.

**Supplementary Information:**

The online version contains supplementary material available at 10.1038/s41598-025-03193-3.

## Introduction

Interest in plant-rich diets, characterized by low to no consumption of foods of animal origin, has increased substantially in recent years due to their potential health benefits and environmental sustainability^1^. With food production being a major source (~ 20–30%) of global greenhouse gas (GHG) emissions^2^ and with a greater focus on mitigating climate change, some public health authorities are now placing stronger emphasis on plant-rich diets in their official recommendations^3,4^.

Although nutritional benefits of plant-rich diets are generally acknowledged, more uncertainty exists around fully-plant-based, or vegan diets. These uncertainties relate to nutritional risks associated with lower bioavailability of certain amino acids in plant-based foods^5^ and lower intakes of nutrients mainly found in animal foods^6^. These risks can, however, easily be mitigated with a diverse diet and/or use of food supplements^7^. There is also some evidence to suggest that adhering to a vegan diet is associated with lower risk of cancer and mortality^8,9^.

According to population-based surveys the prevalence of individuals following vegan diets has traditionally been low (~ 1–2%) and this group has until recently not received much attention in official dietary recommendations^10,11^. Citing lack of data the 2023 Nordic Nutrition Recommendations stated that limited conclusions on nutritional risks and benefits, as well as sustainability, could be drawn from existing studies on vegan diets^3,12^.

To address this data gap, this study aimed to assess possible nutritional health risks and benefits among Icelandic vegans and omnivores. For that purpose, we assessed the compliance of both diets relative to official nutritional recommendations^3^. In addition, the diet of both groups was characterized according to the proportion of energy from ultra-processed foods^13^, which have received considerable attention as a possible determinant of several adverse health outcomes^14^. Furthermore, the environmental sustainability of vegan versus omnivore diets was assessed in terms of dietary greenhouse gas emissions.

## Methods

### Study population

We used data on 651 omnivores and 15 vegans from the Icelandic National Dietary Survey conducted in 2019–2021^15^ combined with data on 53 vegans recruited from a comparable study from 2022 to 2023 through the Icelandic Vegan Association. The National Dietary Survey recruited participants from 18 to 80 years of age but for the purpose of this study we restricted the inclusion to the same age range (18–67 years) as observed among the vegan participants to increase comparability. Both studies collected in an identical manner with detailed information on dietary habits (described below) and participants socio-demographic factors as well as self-reported height and weight. All data collection was performed in compliance with regulations from the Icelandic Data Protection Authority, which includes informed consent from participants, and received ethical approval from the Icelandic National Bioethics Committee (No. VSN-19-115, and VSN-21-159).

### Dietary assessment

For both the National Dietary Survey and the vegan survey, the dietary assessment consisted of two 24-hour recalls and a short food frequency questionnaire to capture the habitual intake of common food groups^12^. The two 24-hour recalls were conducted via phone by trained interviewers on two non-consecutive days, covering weekdays and weekends. Participants also answered several questions, encompassing demographic details, lifestyle, and health. Habitual dietary intake was estimated using the mean of two 24-hour recalls. The 24-hour recalls also recorded the use of dietary supplements. Nutrient intake from foods was assessed by linking the reported amount consumed for individual food items or composite foods (described by recipes) to the Icelandic food composition Table^16^, which contained information on the weight of the edible part of each food and its nutrient content. The loss of water and nutrient content during food preparation was accounted for in our dietary calculation software *FoodCalc v1. 3.*^17^. Similarly, the intake of vitamins, minerals and essential fatty acids from food supplements was estimated by linking the type or brand name of each supplement to the closest available record in the food composition table taking into consideration the amount consumed (i.e. number of pills, capsules or droplets). Consumption of individual foods was also aggregated into different food groups (i.e., meat, dairy, etc., see Table 2) to describe the participant’s habitual dietary intake.

### Dietary habits and compliance with nutritional recommendations

Dietary habits of study participants were described by comparing the mean intake of vegans and omnivores across several food groups as well as comparing their intake for several essential micronutrients. We also compared these intake estimates relative to existing nutritional recommendations using the 2023 Nordic Nutrition Recommendations^3^. For those comparisons we used on the recommended ranges of intake, expressed as percentage of energy (E%), for carbohydrates (45–60%), added and free sugars (< 10%) total fat (25–40%), saturated fat (< 10%) and being above the lower bound for protein intake (≥ 10%). Comparison was also made with the recommended absolute protein intake of ≥ 0.83 g/kg body weight per day and dietary fiber intake of ≥ 25 g and ≥ 35 g/day for females and males, respectively^3^.

Intake of vitamins and minerals were also compared to the recommended intake (RI) or adequate intake (AI) when RI was unavailable, as well as to the estimated average requirement (AR) (see Supplemental Table S1 where RI, AI and AR are also defined). For that comparison median intakes in our study population that fell below AR were interpreted as indications for risk of nutrient deficiency^18^.

### Assessing dietary intake in terms of food processing

We quantified consumption of ultra-processed foods using The NOVA classification system^13^. This was done by adding a grouping variable to the Icelandic food composition table (ISGEM), labeling each food item according to the most relevant NOVA classification group as: Minimally processed food (NOVA group 1), food containing culinary ingredients (NOVA group 2), processed food (NOVA group 3); and ultra-processed food (NOVA group 4). Consumption of unprocessed to ultra-processed foods was then aggregated in terms of percentage of energy intake consumed from each NOVA group.

Although the use of 24-hour recalls has been shown to be reliable when classifying individual foods into NOVA groups^19^, such classification is still subject to some uncertainty. As a result, arbitrary decisions sometimes need to be made when brand names or sources of certain products are unclear. Correct classification of bread, for example, depends on the degree of processing as bread with just a few ingredients, including traditional sourdough and whole wheat bread, belong to NOVA group 3; while industrially produced breads that contain additives, preservatives, emulsifiers, and flavor enhancers are then assigned to NOVA group 4. In cases where decisions on food items (such as bread) were uncertain, a decision was made after discussion by two independent assessors. In the case of dishes that contained ingredients with different levels of processing, the individual components were separated through use of recipes in our calculation software and assigned to the appropriate NOVA classification group.

### Dietary greenhouse gas emissions

Dietary GHG emissions (or carbon footprint) was quantified using the *Big Climate Database* from *CONCITO* Denmark^20^. The database contains GHG emissions estimates for ~ 500 food items commonly found in grocery stores on the European market, including Iceland. The GHG emissions estimates were generated using a hybrid consequential life cycle assessment and input-output analysis, which quantifies GHG emissions (in kg CO_2_-eq) associated with producing one kilogram of each food item.

The GHG emissions estimates from the CONCITO database were linked to the ~ 700 individual and composite foods that participants reported to have consumed in the two 24-hour recalls. This was done by directly linking individual food items in the CONCITO database with the corresponding record in the Icelandic food composition table (ISGEM). When direct linkage could not be made (in approx. 30% of cases), which was mostly related to composite dishes, the corresponding GHG emissions was estimated based on recipes containing the raw ingredients for that composite food items. The total dietary GHG emissions for each participant was then estimated by aggregating the contribution over all food items.

The GHG emissions estimates in the Big Climate database are based on foods sold at the retail level. In our calculations we also accounted for the contribution of food waste and losses in the food supply chain based on expected default losses (in %) as estimated and reported by the Food and Agriculture Organization^21^. Dietary GHG emissions were then reported with and without adjustment for food loss.

Finally, to address concerns raised about inconsistent estimates being reported across databases for the GHG emissions of individual foods^22^, we found no meaningful differences in our dietary GHG emissions estimates among participants in our National Nutrition Survey when comparing the results from the CONCITO database with two other databases from the US and Europe^15^.

### Statistics

Statistical analysis was performed in R and carried out using the R-studio Posit software (version 2024.09.0). Ordinal variables were described using numbers and percentages and differences between groups were formally assessed using the Fischer’s exact or chi-square test. For continuous variables, the mean and standard deviation (SD) were used for normally distributed variables while the median and percentiles (10th and 90th ) were used for skewed continuous variables. Differences between vegans and omnivores were formally evaluated using the *t*-test for normally distributed variables or the Mann-Whitney *U* test for skewed variables. In our analysis we compared mean or median intakes between vegans and omnivores in terms of intake of (1) different food groups, (2) calories from different levels of food processing as defined in the NOVA classification system, (3) essential nutrients and (4) dietary GHG emissions in kg CO_2_-eq/day.

## Results

Characteristics of the study population are presented in Table 1. Those following vegan diets were younger than omnivores (mean: 36 vs. 44 years) and more likely to be females (68% vs. 52%). Vegans also had slightly lower body mass index (BMI) than omnivores (23.7 vs. 26.9 kg/m^2^) and were more likely to have a university education compared to omnivores (75% vs. 52%). Most vegans (97%) and omnivores (72%) reported taking one or more food supplements.

**Table 1:** Characteristics of study population.

	Vegans (*n* = 68)	Omnivores (*n* = 651)	*p*-value ^a^
*Age (year)*,* mean (SD)*	36 (13)	44 (14)	< 0.001
*Age groups*,* n (%)*			
18–29	25 (36%)	128 (20%)	
30–49	29 (43%)	267 (41%)	
50–67	14 (21%)	256 (39%)	
*Females*,* n (%)*	46 (68%)	337 (52%)	0.018
*Highest education*,* n (%)*			< 0.001
Primary School	2 (3%)	68 (10%)	
Upper secondary school	12 (18%)	126 (19%)	
Vocational	1 (1%)	77 (12%)	
University degree	51 (75%)	335 (52%)	
Other	2 (3%)	45 (7%)	
*Smoking*,* n (%)*	2 (3%)	61 (9%)	0.132
*BMI (kg/m*^*2*^*)*,* mean (SD)*	23.7 (4.0)	26.9 (4.7)	< 0.001
*BMI categories*,* n (%)*			
<18.5	3 (5%)	5 (1%)	
18.6-24.99	41 (63%)	241 (38%)	
25-29.99	18 (28%)	253 (39%)	
>30	3 (5%)	141 (22%)	
*Make ends meet* ^*b*^, *n (%)*			0.420
Easy	53 (80%)	479 (74%)	
Difficult	4 (6%)	70 (11%)	
Neither easy nor difficult	9 (14%)	99 (15%)	
*Physically active* ^*c*^, *n (%)*	60 (91%)	547 (84%)	0.194
*Taking food supplements* ^*d*^, *n(%)*	65 (97%)	468 (72%)	< 0.001

Abbreviations: BMI – body mass index.

^a^ P-value for the difference between vegans and omnivores: Fischer exact or chi-square test for categorical variables and the Mann-Whitney *U* test for continuous variables.

^b^ “how difficult or easy it is to make ends meet on a yearly basis”.

^c^ “are you engaged in vigorous exercise for 30 minutes or more per day”.

^d^ “do you take any food supplements (e.g., vitamins, minerals or fish oil supplements)”.

The median (10th, 90th percentile) for intake of different food groups for those following vegan and omnivore diets is shown in Table 2. As expected, those following a vegan diet consumed more plant-rich food groups, including plant-based alternatives to animal-sourced foods. On a few recorded days (i.e., at the 90th percentile) some vegans reported minimal consumption of foods of animal origin including dairy, meat, and animal fats (i.e., lard and butter).

**Table 2:** Dietary intake of vegans and omnivores. The table shows the aggregated median (10th and 90th percentile) consumption from different foods groups in grams per day.

	Vegans (*n* = 68)	Omnivores (*n* = 651)	*p*-value ^a^
	Median intake (10th – 90th percentile)		
Vegetables ^b^	268 (64–457)	141 (49–276)	< 0.001
Fruits	202 (8–491)	59 (0–214)	< 0.001
Cereals and their derivatives	274 (103–399)	162 (66–325)	< 0.001
Plant-based alternatives ^c^	235 (33–576)	0 (0–19)	< 0.001
Legumes	32 (0–136)	0 (0–29)	< 0.001
Nuts, seeds, and dried fruits	23 (0–80)	0 (0–23)	< 0.001
Meat and meat product	0 (0–35)	126 (36–264)	< 0.001
Dairy product	5 (0–315)	600 (212–1287)	< 0.001
Seafood	0 (0–1)	10 (0–124)	< 0.001
Animal fats ^d^	0 (0–32)	0 (0–66)	< 0.001
Vegetable oils	17 (3–54)	9 (1–30)	< 0.001
Confectionery and snack food ^e^	33 (0–102)	57 (0–167)	0.015
Coffee	150 (0–464)	250 (0–674)	0.002
Tea	0 (0–283)	0 (0–100)	< 0.001
Alcoholic beverages	0 (0–493)	0 (0–414)	0.908
Soft drinks, fruit, and vegetable juices	249 (1–852)	328 (0–1057)	0.099
All other foods ^f^	106 (15–228)	35 (7–115)	< 0.001

^a ^P-value for the difference between vegans and omnivores (Mann-Whitney *U* test).

^b ^Including potatoes.

^c ^Plant-based alternatives to animal-sourced foods including soya products and alternative dairy products.

^d ^Lards, tallows, fats, butters, and animal-sourced oils.

^e ^Snacks, sweets, pastry, desserts, chips, and popcorn.

^f ^Including margarine, dressings, sauces, spices, ready meals, and protein products.

The total energy intake, energy from minimally- to ultra-processed foods, and macronutrient composition among vegans and omnivores are presented in Table 3. The percentage of energy intake coming from ultra-processed foods was nearly identical (or ~ 46%) among vegans and omnivores. Differences in food choices between the two groups were mirrored by substantial differences (vegans vs. omnivores) in percentage of energy from macronutrient intake with total dietary fat (35% vs. 40%), saturated fat (11% vs. 16%), and protein (12% vs. 18%) intake being significantly lower among vegans compared to omnivores. However, the percentage of energy from carbohydrates was higher among vegans (48% vs. 29%).

**Table 3:** Distribution of energy and macronutrient intake among vegans and omnivores.

	Vegans (*n* = 68)	Omnivores (*n* = 651)	*p*-value ^a^
	Mean (standard deviation)		
Total energy intake, MJ	9.55 (3.90)	8.78 (2.94)	0.108
%E from minimally processed foods ^b^	29 (14)	32 (13)	0.018
%E from food with culinary ingredients ^b^	5 (4)	6 (5)	0.167
%E from processed foods ^b^	19 (12)	15 (9)	0.019
%E from ultra-processed food ^b^	46 (18)	46 (15)	0.955
Protein, %E	12 (3)	18 (4)	< 0.001
Total fat, %E	35 (7)	40 (7)	< 0.001
Saturated fat, %E	11 (4)	16 (4)	< 0.001
Carbohydrate, %E	48 (8)	39 (8)	< 0.001
Fibres from cereals, g	9 (5)	7 (4)	< 0.001
Fibres from other sources, g	28 (19)	11 (6)	< 0.001
Added sugar, %E	6 (7)	8 (6)	< 0.001

^a ^P-value for the difference between vegans and omnivores (Mann-Whitney *U* test).

^b ^Based on the NOVA classification system^15^.

**Fig. 1:**
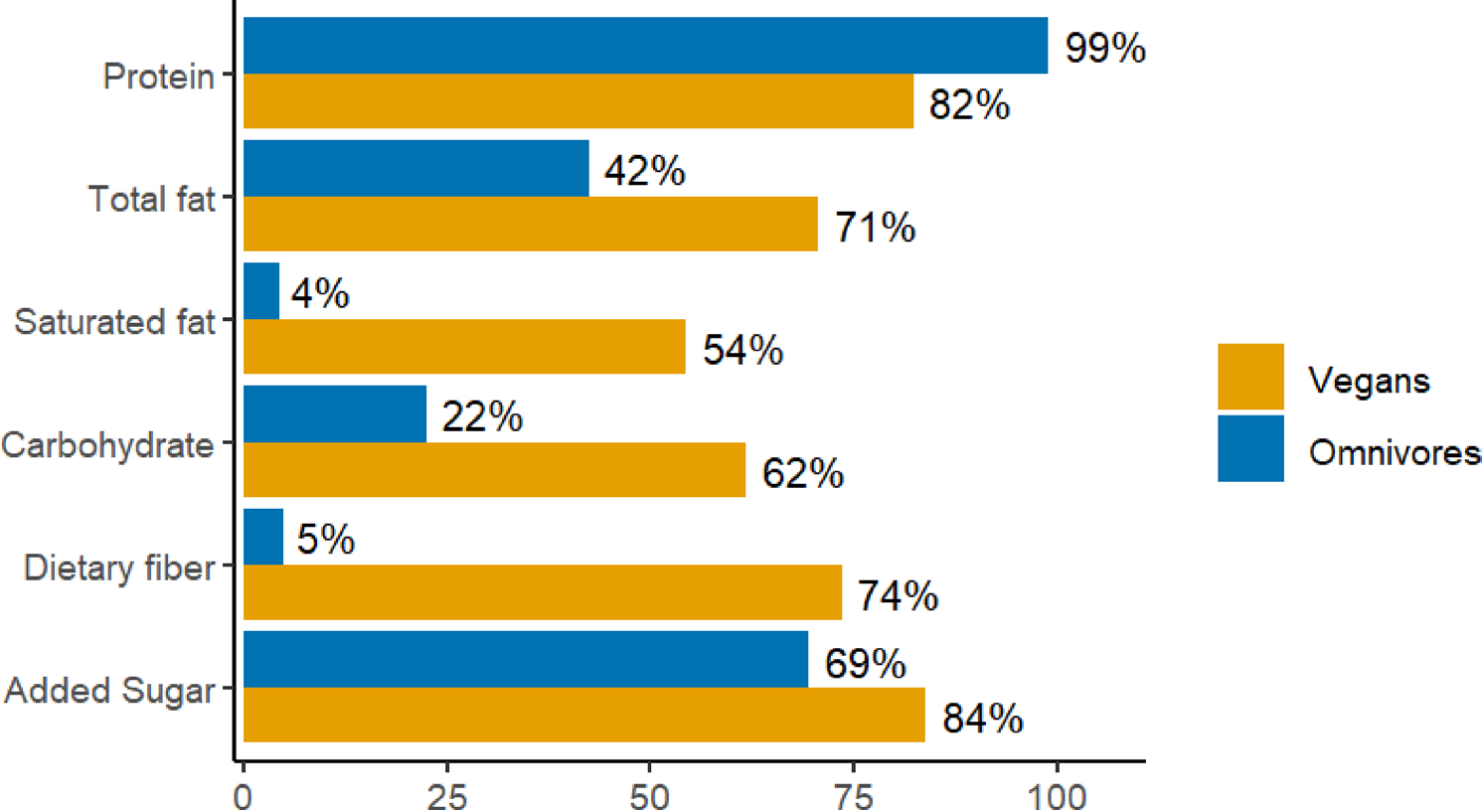
Percentage of vegans and omnivores within the ranges of the dietary reference values for macronutrients as reported in the 2023 Nordic Nutrition Recommendations^4^. These reference values are expressed in terms percentage intake of total energy (%E) for proteins (≥ 10%E), total fats (25–40%E), saturated fats (< 10%E), carbohydrates (45–60%), added sugars (< 10%E); but absolute intakes for fibers (females > 25 g/day, males > 35 g/day).

Vegans compared to omnivores were also more likely to meet the recommendation for the proportion of energy intake from carbohydrates, fibers, added sugars, total fat and saturated fat (see Fig. 1). As shown in Fig. 1; Table 4, vegans, compared to omnivores, were less likely to meet protein recommendations both when expressed in terms of having protein intake equal to or above 10% of energy intake (82% vs. 99%) or when expressed as intake of ≥ 0.83 g protein/kg body weight (52% vs. 79%).

**Table 4:** Combined intake of selected vitamins and minerals intakes from food and food supplements among vegans and omnivores. For each nutrient estimated intake is compared with values for recommended intake/adequate intake and average requirement ^a^.

Nutrients	Vegans (*n* = 68)			Omnivores (*n* = 651)		
	Median intake (10th – 90th percentile)	Above RI/AI	Above AR	Median intake (10th – 90th percentile)	Above RI/AI	Above AR
Vitamin D, µg	11.0 (0.4–56.3)	54%	56%	11.2 (1.8–51.6)	52%	58%
Vitamin B6, µg	3.0 (1.2–11.9) ^*^	79%	88%	2.4 (1.1–9.6)	68%	80%
Folate, µg	484 (189–1095) ^*^	68% ^*^	79%	296 (152–678)	43%	61%
B12 Vitamin, µg	3.5 (0.7–9.7) ^*^	41% ^*^	56% ^*^	6.3 (2.7–13.2)	77%	85%
Vitamin C, mg	174 (64–424) ^*^	76% ^*^	85% ^*^	83 (24–270)	40%	51%
Calcium (Ca), mg	701 (406–1311) ^*^	28% ^*^	40% ^*^	897 (437–1605)	44%	62%
Iron (Fe), mg	16.7 (9.7–38.5) ^*^	76% ^*^	93% ^*^	9.7 (5.3–19.8)	46%	71%
Iodine (I), µg b	74 (13–213) ^*^	25% ^*^	38%	127 (50–331)	41%	53%
Sodium (Na), mg	3.0 (1.4–4.6)	38% ^*^	-	3.0 (1.9–4.8)	23%	-
Protein, g	62 (40–103) ^*^	-	-	88 (55–137)	-	-
Protein, g/kg body weight	0.87 (0.51–1.69) ^*^	52% ^*^	74% ^*^	1.10 (0.68–1.75)	79%	91%

Abbreviations: RI - recommended intake, AI – adequate intake, AR – average requirement.

^a ^AI is used when an RI has not been established in 2023 NNR.

^*^Significant differences between vegans and omnivores in nutrient intake (Mann-Whitney *U* test); or proportion above RI/AI or proportion above AR (chi-square test).

After accounting for the intake of food supplements (Table 4), the median intake of calcium (701 vs. 897 mg/day) and iodine (74 vs. 127 µg/day) were lower among vegans compared to omnivores and for vegans the median was in both cases below the AR. On the other hand, intake of vitamin C (174, vs. 82 mg/day), folate (484 vs. 296 µg/day) and iron (16.7 vd 9.7 mg/day ws substantially higher among vegans compared to omnivores. In other cases, no major differences were observed in the proportion of vegans and omnivores reaching above the average requirements for individual micronutrients. Intakes of these micronutrients excluding the contribution of food supplements are shown in Supplemental Table S2.

**Fig. 2:**
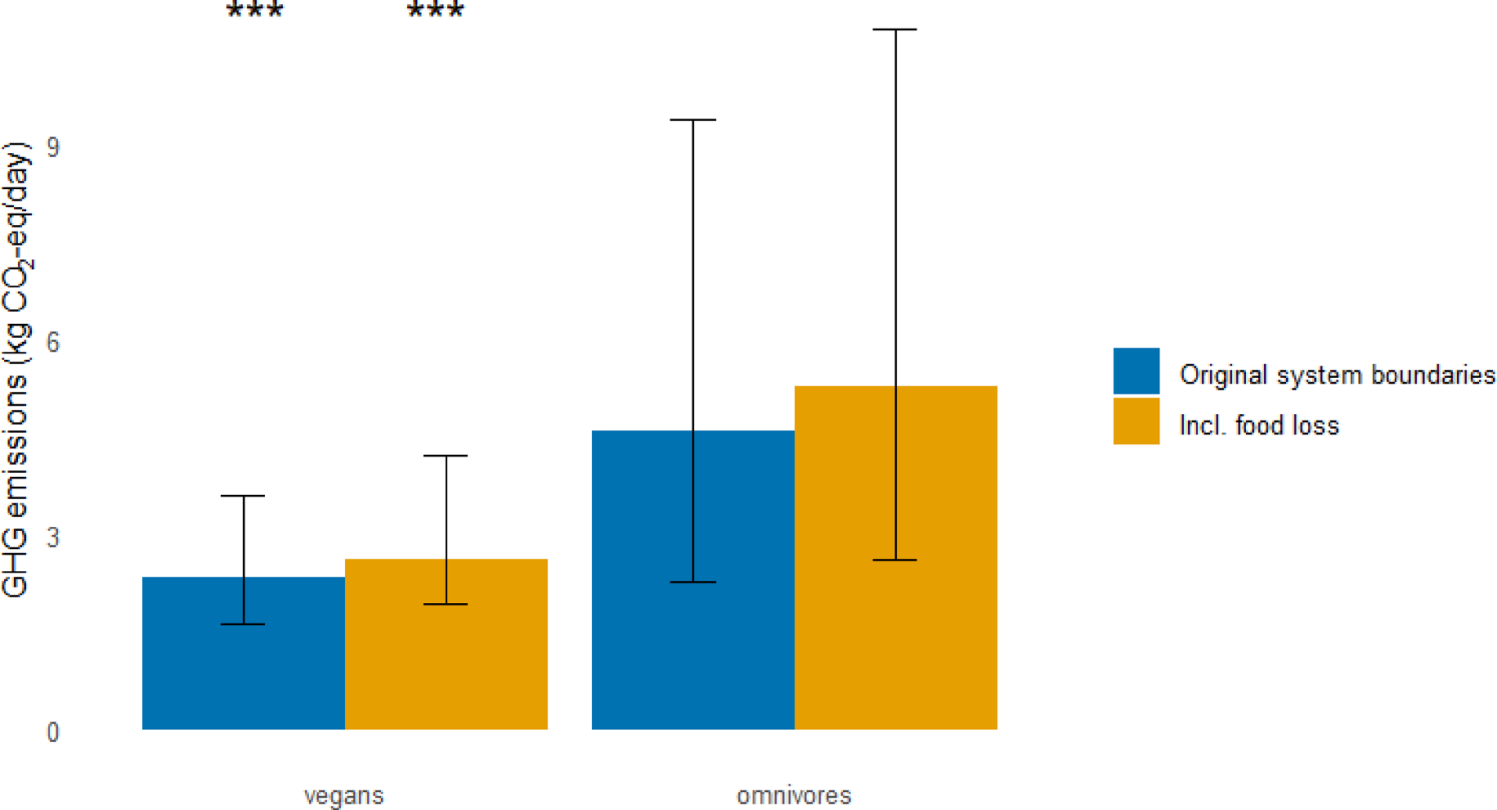
The median (10th – 90th percentile) dietary greenhouse gas emissions of vegan and omnivore diets. The blue bars show the results based on the original system boundaries for the CONCITO database^17^ while for the orange bars contribution of food loss up to the retail level has been added. ^***^ Indicates significant difference between vegans and Omnivores (Mann-Whitney *U* test. *P* < 0.001).

After adjustment for food loss up to retail level, the median (10th, 90th percentile) total dietary GHG emissions from those adhering to vegan diet were 2.6 kg CO_2_-eq/ day (1.9, 4.2) compared to 5.3 kg CO_2_-eq/day (2.6, 10.8) for omnivores (Fig. 2). The higher GHG emissions among omnivores were mostly driven by meat and dairy consumption (see Supplemental Table S3 and S4, where the exact numbers for total dietary GHG emissions in Fig. 2 and contribution from each food group are shown). Differences in total dietary GHG emissions could not be explained by differences in energy intake which was modest (~ 10%) between the two groups (see Table 3).

## Discussion

In this study, those adhering to vegan diets had twofold lower dietary GHG emissions than omnivores. Except for protein intake, dietary habits of vegans were more in line with recommendations for macronutrients^3^. Compared to omnivores, vegans were also more in line with recommendations for several micronutrients although some indications of insufficient intake of calcium and iodine were observed. Overall, it was observed that both groups could improve their intake of micronutrients. Our results suggest that more explicit guidance is needed for individuals adhering to plant-rich diets within food-based dietary guidelines, which often do not address how to achieve optimal nutrient intake among those consuming little to no animal-sourced foods.

Compared to omnivores, our vegan participants were younger, had higher education, and were more likely to be females. The higher proportions of females in our study reflects the gender distribution within the Icelandic Vegan Association from which most vegan participants were recruited. Interestingly, few vegans reported on some day’s minimal consumption of animal- sourced foods as reflected by the 90th intake percentile (see Table 2). One explanation might be that some processed foods such as confectionary products can contain small amounts of dairy and animal fats, which consumers may not be aware of^23^. In addition, some vegans may, on rare occasions, intentionally choose to consume foods of animal origin for practical- or social reasons. In that context, it is worth noting that there is no clear consensus on the definition of veganism^24^. For example, in the definition of veganism by the UK Vegan Society the consumption of foods of animal origin is partly acknowledged with the formulation “*seeks to exclude*,* as far as is possible and practicable*”^25^ while the definition by The American Vegan society does not allow for such flexibility^26^. Our results suggest that vegans in our study may have adopted a more flexible approach.

In our study, the total dietary GHG emissions among vegans was twofold lower compared to omnivores. Although such differences have been observed in other studies^27,28^, more varying estimates have also been reported^29^. This may occur due to unaccounted differences in energy intake, use of different environmental databases, or variation in dietary habits^29^. In our study energy intake among vegans and omnivores was within 10% and could not explain the differences observed. Furthermore, in a separate study, we have assessed the impact of using different databases to assess dietary GHG emissions for all participants in the Icelandic National Dietary Survey^15^. The resulting dietary GHG emissions estimates were within 10% in all cases. It therefore appears unlikely that our results are confounded by energy intake or choice of environmental database. Furthermore, our results are also in line with the growing literature which clearly shows that vegans compared to omnivore diets have substantially lower dietary GHG emissions^27,28,30^.

Overall, the dietary habits of vegans were more compliant in relation to macronutrient recommendations^3^. That is, carbohydrate quality was generally higher as reflected by higher proportion of vegans meeting recommendations for total carbohydrates, dietary fiber, and added sugars. The total fat and saturated fat intake of vegans was also more in line with recommendations. In terms of debates on whether there is sufficient evidence to relax the current recommendation for saturated fat intake^31,32^, it is noteworthy that even among vegans in our study only 54% complied with this recommendation while the corresponding number of omnivores was only 4%.

On the other hand, fewer vegans complied with the recommended intake for protein^3^. Lower protein intake among vegans compared to omnivores is also commonly observed in other studies^29–31^. Despite lower protein intake among vegans, the median intake (62 g/day or 0.87 g/kg body weight) was well above the estimated average requirement for adults (0.66 g/kg body weight), which does not raise any major concern in terms of protein deficiency. However, such intake may still lead to increased vulnerability during sensitive life stages such as during pregnancy and older age when protein requirement increases^3,33^. To the best of our knowledge, no studies among older adults adhering to vegan diets are available. However, in the case of pregnant women a study comparing birth outcomes among omnivores, vegetarians, and vegans observed around 200 g lower birth weight of infants among vegan compared to omnivore mothers^34^. In that study the mean intake of protein among vegans was around 10% of total energy which is slightly lower than the 12% observed in our study.

Concerning food processing our findings that consumption of ultra-processed foods was comparable (~ 46% of energy intake) among vegans and omnivores seems to reflect the growing availability of processed plant-rich foods for vegans^35^. Although the NOVA classification system has its limitations^36^, it does provide additional information on the type and source of the food consumed. This in combination with assessment of nutrient intake provides a more complete picture of the participants’ dietary habits.

When quantifying combined micronutrient intake from food and supplements, the median intake among vegans and omnivores only slightly exceeded the average requirement for several nutrients. For vegans, the median intake of calcium and iodine was below the average requirement, which may suggest some risk of nutritional insufficiency^18^. Similar indications have also been observed in a small study on vegans from Germany^37^. In the Nordic countries and the UK, iodine deficiency has been improved either through iodized salt and/or iodine fortification of cow’s fodder to increase the iodine content of dairy^38,39^. The indirect fortification of dairy does, for obvious reasons, not benefit vegans but similar measures for other foods could in the same way improve their intake^40^. This also applies to calcium where dairy alternatives are not consistently fortified^41^. In the absence of such measures, more emphasis on specific supplemental use among vegans seems warranted.

In terms of limitations, our results on micronutrient intake should be interpreted with some caution as the use of a two-day 24-hour recall may inflate the number of subjects estimated to have both low and high intakes^42^ and accurate quantification of nutrients from food supplements can be challenging^43^. This combined with the relatively small sample size of vegans in our study (*n* = 68) is a source of uncertainty, particularly when trying to estimate the prevalence of those within and outside certain dietary reference values. On the other hand, the strength of our study lies in identical assessment and comparison between vegans and omnivores, characterizing their intake in terms of nutritional guidelines and assessing the dietary carbon footprint. Such direct comparisons with official recommendations have been largely lacking, at least in the Nordic setting^3^.

In conclusion, we observed that those adhering to vegan diets were overall more compliant with meeting macronutrient recommendations compared to omnivores and had substantially lower dietary GHG emissions. When evaluating nutrient intake from food and dietary supplements, possibilities for improvements in terms of meeting recommendations were observed in both groups. Our results suggest that food-based dietary guidelines should differentiate between vegans and omnivores, considering the varying nutritional risks that may be associated with each diet. Our results also indicate that in our omnivore population a shift towards a more plant-rich diet without eliminating all foods of animal origin could lead to substantial nutritional and environmental benefits.

## Electronic supplementary material

Below is the link to the electronic supplementary material.

Supplementary Material 1

## Data Availability

Data will be made available on request. Contact the corresponding author.

## Abbreviations

AI: Adequate intake
BMI: Body mass index
E%: Percentage of energy intake
GHG: Greenhouse gas
RI: Recommended intake
