# Supplementary material for: Vegan and omnivore diets in relation to nutrient intake and greenhouse gas emissions in Iceland
Source: Sci Rep. 2025 May 25;15:18190. doi: 10.1038/s41598-025-03193-3 (PMC12104359; doi:10.1038/s41598-025-03193-3)
Supplement: Supplementary file 1 — Supplementary Material 1 [file 41598_2025_3193_MOESM1_ESM.docx]

**Vegan and omnivore diets in relation to nutrient intake and greenhouse gas emissions in Iceland**

Ragnhildur Gudmannsdottir^1, 2*^, Steina Gunnarsdottir^1^, Emese Kenderesi^1^, Holmfridur Thorgeirsdottir^3^, Johanna Eyrun Torfadottir^3, 4^, Ingibjorg Gunnarsdottir^1, 2^, Inga Thorsdottir^1,2,7^, Amanda Wood^5^, Olof Gudny Geirsdottir^1, 2^, Bryndis Eva Birgisdottir^1, 2^, Thorhallur Ingi Halldorsson^1, 6^

**Definition of AR, RI and AI as defined in the 2023 Nordic Nutrition Recommendations in Table 5 [1]:**

**Average Requirement (AR):** *“The average daily nutrient intake level that is estimated to meet the requirements of half of the individuals in a particular life-stage group in the general population. AR is usually used to assess adequacy of nutrient intake of groups of people, and may be used in planning for groups.”*

**Recommended Intake (RI):** *“The average daily dietary nutrient intake level that is sufficient to meet the nutrient requirements of nearly all (usually 97.5%) individuals in a particular life-stage group in the general population. It can be used as a guide for daily intake by individuals. Usually used to plan diets for groups and individuals.”*

**Adequate Intake (AI):** *“The recommended average daily intake level based on observed or experimentally determined approximations or estimates of nutrient intake by a group people that are assumed to be adequate. The AI has larger uncertainty than RI. Can be used when an RI cannot be determined. The AI is expected to meet or exceed the needs of most individuals in a life-stage group.”*

1. Blomhoff, R., et al., *Nordic Nutrition Recommendations 2023 : Integrating Environmental Aspects*. Nord. 2023, Copenhagen: Nordisk Ministerråd. 388 (2023)

**Supplementary Table S1:** Dietary reference values for selected nutrients as reported in the 2023 Nordic Nutrition Recommendations ^a^. For Micronutrients adequate intake (AI) is used when recommended intake (RI) is not available.

|  | Females | | Males | |
| --- | --- | --- | --- | --- |
| *Nutrients* | RI/AI | AR | RI/AI | AR |
| Vitamin D, μg | 10 | 7.5 | 10 | 7.5 |
| B6 Vitamin, mg | 1.6 | 1.3 | 1.8 | 1.5 |
| Folate, μg | 330 | 250 | 330 | 250 |
| B12 Vitamin ^b^, μg | 4 | 3.2 | 4 | 3.2 |
| Vitamin C, mg | 95 | 75 | 110 | 90 |
| Calcium (Ca), mg ^c^ | 950 | 750 | 950 | 750 |
| Iron (Fe), mg ^d^ | 15 | 9 | 9 | 7 |
| Iodine (I) ^b^, μg | 150 | 120 | 150 | 120 |
| Sodium (Na), g | 2.3 | - | 2.3 | - |
| Protein, g/kg | 0.83 | 0.66 | 0.83 | 0.66 |

Abbreviations: RI – recommended intake, AI – adequate intake, AR – average requirement

^a^ Blomhoff, R., et al., *Nordic Nutrition Recommendations 2023 : Integrating Environmental Aspects*. Nord. 2023, Copenhagen: Nordisk Ministerråd. 388.

^b^ AI is used when an RI has not bene established in 2023 NNR

^c^ Values for Ca in the table apply to adults >24 years old

^d^ Values for Fe in the table apply to adults ≥18 and ≤50 years old

**Supplementary Table S2:** Intake of selected vitamins and minerals from food (excluding food supplements) among vegans and omnivores

|  |  |  |
| --- | --- | --- |
|  | **Vegans (n = 68)** | **Omnivores (n = 651)** |
| *Nutrients* | *Median intake (10^th^ – 90^th^ percentile) ^a^* | |
| Vitamin D, μg | 2.2 (0.2 – 6.7) | 3.9 (1.1 – 10.0) |
| Vitamin B6, μg | 2.0 (1.1 – 8.2) | 1.7 (0.9 – 5.1) |
| Folate, μg | 324 (157 – 681) | 235 (140 – 394) |
| B12 Vitamin, μg | 1.4 (0.2 – 6.) | 5.1 (2.3 – 11.2) |
| Vitamin C, mg | 125 (40 – 275) | 69 (21 – 159) |
| Calcium (Ca), mg | 689 (406 – 1311) | 880 (424 – 1551) |
| Iron (Fe), mg | 13.3 (7.6 – 24.6) | 8.9 (5.0 – 14.3) |
| Iodine (I), μg | 44 (12 – 151) | 112 (49 – 290) |
| Sodium (Na), g | 3.0 (1.4 – 4.6 ) | 3.0 (1.9 – 4.9) |
|  |  |  |

^a^ All variables were significantly different between the diets apart from Sodium, assessed using the Mann-Whitney *U* test (p value < 0.05)

**Supplementary Table S3**: Total dietary GHG emissions (*in kg CO_2_-eq/day)* and absolute contribution from different food groups among vegans and omnivores with contribution from food waste and losses up to the retail level

|  | **Vegans (n= 68)** |  | **Omnivores (n= 651)** |
| --- | --- | --- | --- |
|  | *Median dietary GHG emissions (10^th^ and 90^th^ percentile) ^a^* | | |
| Vegetables ^b^ | 0.34 (0.08 – 0.67) |  | 0.17 (0.05 – 0.41) |
| Fruits | 0.30 (0.01 – 0.78) |  | 0.09 (0.00 – 0.33) |
| Cereals and their derivates | 0.38 (0.15 – 0.62) |  | 0.22 (0.08 – 0.48) |
| Plant-based alternatives ^c^ | 0.21 (0.03 – 0.56) |  | 0.00 (0.00 – 0.02) |
| Legumes and pulses | 0.06 (0.00 – 0.23) |  | 0.00 (0.00 – 0.05) |
| Nuts, seeds, and dried fruit | 0.09 (0.00 – 0.28) |  | 0.00 (0.00 – 0.09) |
| Meat and meat products | 0.00 (0.00 – 0.36) |  | 2.26 (0.18 – 7.73) |
| Dairy products | 0.01 (0.00 – 0.33) |  | 0.58 (0.23 – 1.19) |
| Seafood | 0.00 (0.00 – 0.01) |  | 0.10 (0.00 – 2.07) |
| Animal-sourced fats ^d^ | 0.00 (0.00 – 0.02) |  | 0.00 (0.00 – 0.12) |
| Vegetable fat | 0.05 (0.01 – 0.16) |  | 0.03 (0.00 – 0.11) |
| Confectionery and snack food ^e^ | 0.11 (0.00 – 0.35) |  | 0.16 (0.00 – 0.49) |
| Coffee | 0.08 (0.00 – 0.23) |  | 0.13 (0.00 – 0.34) |
| Tea | 0.00 (0.00 – 0.06) |  | 0.00 (0.00 – 0.02) |
| Alcoholic beverages | 0.00 (0.00 – 0.40) |  | 0.00 (0.00 – 0.40) |
| Soft drinks, fruit, and vegetable juice | 0.23 (0.00 – 0.75) |  | 0.21 (0.00 – 0.68) |
| All other food ^f^ | 0.21 (0.02 – 0.46) |  | 0.10 (0.01 – 0.30) |
| Total dietary GHG emissions | 2.60 (1.94 – 4.23) |  | 5.28 (2.62 – 10.80) |

Abbreviations: GHG – greenhouse gas

^a^ All food groups were significantly different between the diets apart from “Coffee,” “Tea,” “Alcoholic beverages,” “Soft drinks, fruit, and vegetable juice” (Mann-Whitney *U* test)

^b^ Including potatoes

^c^ Plant-based alternatives to animal-sourced foods including soya and alternative dairy products

^d^ Lards, tallows, fats, butters, and animal-sourced oils

^e^ Snacks, sweets, pastry, desserts, chips, and popcorn

^f^ Including margarine, dressings, sauces, spices, ready meals, and protein products

**Supplementary Table S4**: Total dietary GHG emissions and absolute contribution from different food groups among vegans and omnivores without contribution from food waste and losses up to the retail level (that is the original system boundaries of the CONCITO database)

|  | **Vegans (n= 68)** |  | **Omnivores (n= 651)** |
| --- | --- | --- | --- |
|  | *Median dietary GHG emissions (10^th^ and 90^th^ percentile) ^a^* | | |
| Vegetables ^b^ | 0.23 (0.05 – 0.42) |  | 0.11 (0.04 – 0.25) |
| Fruits | 0.21 (0.01 – 0.53) |  | 0.06 (0.00 – 0.22) |
| Cereals and their derivates | 0.31 (0.12 – 0.51) |  | 0.18 (0.7 – 0.39) |
| Plant-based alternatives ^c^ | 0.21 (0.03 – 0.56) |  | 0.00 (0.00 – 0.02) |
| Legumes and pulses | 0.05 (0.00 – 0.19) |  | 0.00 (0.00 – 0.04) |
| Nuts, seeds, and dried fruit | 0.08 (0.00 – 0.24) |  | 0.00 (0.00 – 0.08) |
| Meat and meat products | 0.00 (0.00 – 0.32) |  | 1.98 (0.16 – 6.79) |
| Dairy products | 0.01 (0.00 – 0.31) |  | 0.55 (0.22 – 1.12) |
| Seafood | 0.00 (0.00 – 0.01) |  | 0.07 (0.00 – 1.60) |
| Animal-sourced fats ^d^ | 0.00 (0.00 – 0.02) |  | 0.00 (0.00 – 0.12) |
| Vegetable fat | 0.05 (0.01 – 0.15) |  | 0.03 (0.00 – 0.11) |
| Confectionery and snack food ^e^ | 0.11 (0.00 – 0.34) |  | 0.16 (0.00 – 0.48) |
| Coffee | 0.08 (0.00 – 0.23) |  | 0.13 (0.00 – 0.34) |
| Tea | 0.00 (0.00 – 0.06) |  | 0.00 (0.00 – 0.02) |
| Alcoholic beverages | 0.00 (0.00 – 0.40) |  | 0.00 (0.00 – 0.40) |
| Soft drinks, fruit, and vegetable juice | 0.23 (0.00 – 0.75) |  | 0.21 (0.00 – 0.68) |
| All other food ^f^ | 0.21 (0.02 – 0.46) |  | 0.10 (0.01 – 0.30) |
| Total dietary GHG emissions | 2.33 (1.62 – 3.61) |  | 4.60 (2.27 – 9.39) |

Abbreviations: GHG – greenhouse gas

^a^ All food groups were significantly different between the diets apart from “Coffee,” “Tea,” “Alcoholic beverages,” “Soft drinks, fruit, and vegetable juice” (Mann-Whitney *U* test)

^b^ Including potatoes

^c^ Plant-based alternatives to animal-sourced foods including soya and alternative dairy products

^d^ Lards, tallows, fats, butters, and animal-sourced oils

^e^ Snacks, sweets, pastry, desserts, chips, and popcorn

^f^ Including margarine, dressings, sauces, spices, ready meals, and protein products
